# The metabolites from traditional Chinese medicine targeting ferroptosis for cancer therapy

**DOI:** 10.3389/fphar.2024.1280779

**Published:** 2024-07-03

**Authors:** Yu Tang, Ying Zhuang, Chuanxiang Zhao, Shuangshuang Gu, Junya Zhang, Shiqi Bi, Ming Wang, Lei Bao, Mei Li, Wei Zhang, Liqun Zhu

**Affiliations:** ^1^ Department of Pathology, Affiliated Hospital of Jiangsu University, Zhenjiang, Jiangsu, China; ^2^ Institute of Medical Genetics and Reproductive Immunity, School of Medical Science and Laboratory Medicine, Jiangsu College of Nursing, Huai’an, Jiangsu, China; ^3^ Shanghai Institute of Rheumatology, Shanghai Renji Hospital, Shanghai Jiaotong University School of Medicine, Shanghai, China; ^4^ Department of Gastroenterology, Affiliated Hospital of Jiangsu University, Zhenjiang, Jiangsu, China; ^5^ Department of Medical Imaging, Affiliated Hospital of Jiangsu University, Zhenjiang, Jiangsu, China

**Keywords:** botanical drugs, ferroptosis, neoplasm, phytomedicine, traditional Chinese medicine

## Abstract

Cancer is a major disease with ever-increasing morbidity and mortality. The metabolites derived from traditional Chinese medicine (TCM) have played a significant role in combating cancers with curative efficacy and unique advantages. Ferroptosis, an iron-dependent programmed death characterized by the accumulation of lipid peroxide, stands out from the conventional forms of cell death, such as apoptosis, pyroptosis, necrosis, and autophagy. Recent evidence has demonstrated the potential of TCM metabolites targeting ferroptosis for cancer therapy. We collected and screened related articles published in or before June 2023 using PubMed, Google Scholar, and Web of Science. The searched keywords in scientific databases were ferroptosis, cancer, tumor, traditional Chinese medicine, botanical drugs, and phytomedicine. Only research related to ferroptosis, the metabolites from TCM, and cancer was considered. In this review, we introduce an overview of the current knowledge regarding the ferroptosis mechanisms and review the research advances on the metabolites of TCM inhibiting cancer by targeting ferroptosis.

## 1 Introduction

In the past decades, the global morbidity of cancer has increased, and cancers have become the leading cause of mortality worldwide. According to the latest statistics from the International Agency for Research on Cancer (IARC), there were 19.3 million new cancer cases and 10.0 million deaths globally in 2020 (Sung et al., 2021). Current knowledge has established that the ability of cancer cells to evade cell death is the key feature of tumorigenesis. Nowadays, the regimens of cancer therapy, such as surgery, chemotherapy, radiotherapy, targeted therapy, and immunotherapy, aim to induce the death of cancer cells to achieve a therapeutic effect, whereas the intrinsic and acquired resistance of cancer cells to cell death often results in far from satisfactory treatment outcomes (Loftus et al., 2022; Wang et al., 2022).

Ferroptosis is a recently discovered form of cell death that is distinct from other forms at the morphological, biological, and genetic levels. This unique form of cell death is an iron- and reactive oxygen species (ROS)-dependent form of programmed cell death that is controlled by integrated oxidation and antioxidant systems (Dixon et al., 2012). Numerous studies have confirmed that ferroptosis is an important player in cancer biology and therapies. Several clinical drugs approved by the U.S. Food and Drug Administration (FDA), such as sorafenib, sulfasalazine, and artesunate, can induce ferroptosis in several cancer cells via inhibiting system Xc^−^ (Lachaier et al., 2014; Lu et al., 2017; Li Z.-j. et al., 2020). The sulfasalazine-induced ferroptosis is partially eliminated by efficient ferroptosis inhibitors, supporting the rationale of applying ferroptosis to treat cancer in preclinical and clinical settings (Yin et al., 2023). Accordingly, there is an urgent need to discover ferroptosis-inducing drugs for the treatment of cancers, especially drug-resistant cancers.

Traditional Chinese medicines (TCMs) have a long history and play an important role in treating cancers. Many metabolites of TCM have been used for prophylaxis and therapeutics of cancers with unique advantages (Xiang et al., 2019). These metabolites reverse cancer multidrug resistance, reduce postoperative adverse reactions, improve patients’ quality of life, and potentiate anti-PD-1/PD-L1 immunotherapy (Huang F. et al., 2022; Wei et al., 2022; Qin et al., 2023). However, the exact targets and signal pathways of most TCM drugs used in the treatment process are not fully elucidated. The exploration of the mechanism of the metabolites from TCM drugs in cancer prevention and treatment, or optimization of good anti-tumor drugs from TCM drugs, has become a major research focus. In this review, we illustrate the molecular mechanism of ferroptosis and its regulation, highlight the role of metabolites of TCMs targeting ferroptosis to treat cancer, and discuss the challenges in cancer therapy.

## 2 Molecular mechanisms of ferroptosis

In 2003, the Stockwell Lab identified a non-apoptotic form of cell death when they used the small molecule erastin to stimulate the oncogenic RAS-mutant human cancer cell line while screening anticancer drug libraries (Dolma et al., 2003). In 2008, this lab reported that an additional complex RAS synthetic lethal 3 (RSL3) induced a similar cell death form, which could be inhibited by iron chelates (Yang and Stockwell, 2008). Based on these findings, Brent R. Stockwell and others officially introduced the concept of ferroptosis to describe the iron-dependent cell death driven by lipid peroxidation on the plasma membranes of cells (Dixon et al., 2012).

**FIGURE 1:**
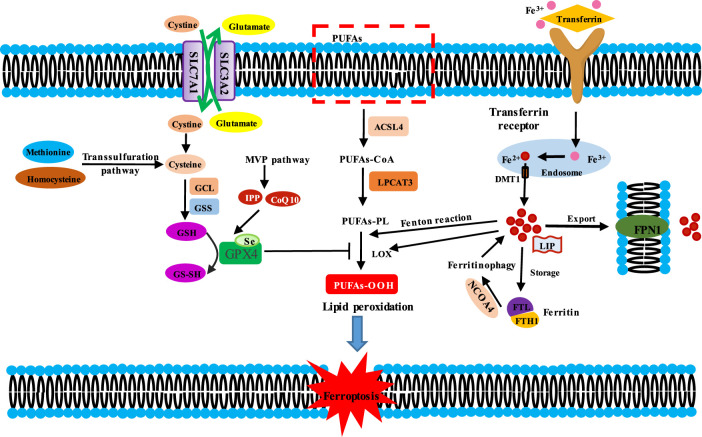
Molecular mechanisms of ferroptosis induction. Induction mechanisms of ferroptosis are divided into three kinds. Ferroptosis is initiated by two key signals, i.e., the inhibition of antioxidant system Xc^−^-glutathione (GSH)-glutathione peroxidase 4 (GPX4) and the accumulation of iron. Cysteine is transported into the cells by SLC7A11/SLC3A2 complex for GSH synthesis. GSH is the substrate for GPX4, which reduces lipid hydroperoxides (L-OOH) to the corresponding alcohol (L-OH) or free H_2_O_2_ to water, thereby preventing lipid peroxidation. Thus, the inhibition of antioxidant system Xc^−^-GSH-GPX4 triggeres ferroptosis. Fe^3+^ is bound with transferrin (TF) to form TF-Fe^3+^ complex, then which could be endocytosed through the cell membrane transferrin receptor 1 (TFR1) into cell. In the cells, Fe^3+^ is reduced to Fe^2+^ in the endosome, which is transported out by divalent metal ion transporter 1 (DMT1), and then is involved in the unstable iron pool (LIP). Fe^2+^ in LIP could be stored in ferritin and is pumped out of cell by ferritin 1 (FPN1). Besides, ferritin could be degraded in lysosome by ferritinophagy. Fe^2+^ mediates Fenton reaction and functions as a cofactor for lipoxygenases (LOXs), thus triggering lipid peroxidation. The peroxidation of PUFAs is the excution of ferroptosis. ACSL4 catalyzes the ligation of long-chain PUFAs with CoA, and LPCAT3 promotes the esterification and incorporation of these products into membrane phospholipids (PL). PUFA-containing PL is oxidized by iron-dependent enzymes LOX or iron-mediated Fenton reaction, leading to lipid peroxidation, membrane damage and subsequent ferroptosis.

Ferroptosis differs from other forms of cell death, such as apoptosis, necroptosis, pyroptosis, and autophagy. Morphologically, the mitochondria are smaller with an increase in mitochondrial membrane density and reduction or disappearance of mitochondrial cristae as well as rupture of the outer mitochondrial membrane, but the nuclear size is intact (Dolma et al., 2003; Dixon et al., 2012). Biochemically, the accumulation of iron-dependent lipid peroxides and ROS leads to membrane instability and even rupture, eventually triggering ferroptosis (Chen et al., 2021b). The main mechanisms of ferroptosis can be characterized in three aspects: the inhibition of the system X_c_^−^-glutathione (GSH)–glutathione peroxidase 4 (GPX4) axis, the elevation of iron metabolism axis, and the lipid metabolism (Figure 1).

### 2.1 The inhibition of the system Xc^−^-glutathione (GSH)–glutathione peroxidase 4 (GPX4) axis

The system Xc^−^ is a heterodimeric antiporter on the plasma membrane, which is composed of solute carrier family seven-member 11 (SLC7A11/xCT) and solute carrier family three member 2 (SLC3A2) with a disulfide bond (Sato et al., 1999). The system Xc^−^ functions to exchange extracellular cystine and intracellular glutamate at a ratio of 1:1 (Dixon et al., 2012). In the cell, cystine is reduced to cysteine, which is a precursor for GSH synthesis. GSH is an indispensable cofactor for the function of GPX4, which reduces lipid hydroperoxides (L-OOH) to the corresponding alcohol (L-OH) or free H_2_O_2_ to water (Dixon et al., 2012). Thus, the inhibition of the system Xc^−^–GSH-GPX4 axis leads to the accumulation of lethal lipid peroxides and the occurrence of ferroptosis.

Inhibition of system Xc^−^ (inhibiting SLC7A11 expression) influences the GSH synthesis, which decreases GPX4 activity and ultimately triggers ferroptosis. In the case of iron overload, the loss of SLC7A11 contributes to ferroptosis-related liver injury (Wang et al., 2017). Moreover, the small molecules erastin and sorafenib could block system Xc^−^ function, triggering ferroptosis in cancer cells derived from the liver, kidney, and lung (Louandre et al., 2013; Dixon et al., 2014; Lachaier et al., 2014). In addition, P53 could downregulate the SLC7A11 expression to inhibit cystine uptake, thereby affecting GPX4 activity and ultimately promoting the occurrence of ferroptosis (Jiang et al., 2015). However, SLC7A11 KO mice were normal in appearance and fertile *in vivo*, whereas SLC7A11^−/−^ embryonic fibroblasts underwent cell death in routine culture medium *in vitro* but could proliferate normally in the presence of 2-mercaptoethanol (2ME), N-acetyl cysteine (NAC), or vitamin E (Stipanuk, 2004; Sato et al., 2005). The difference could be attributed to the absorption of cystine (or cysteine) or acquisition of intracellular cysteine by the additional transport systems to compensate for the loss of SLC7A11. This can be exemplified in the case of homocysteine. Homocysteine could be converted into cystathionine (the precursor of cysteine), and thus, cysteine was supplemented via the trans-sulfuration pathway (McBean, 2012). The deletion of cysteinyl-tRNA synthetase (CARS) resulted in the accumulation of cystathionine and inhibited the elevation of erastin-induced lipid reactive oxygen species, thus suppressing ferroptosis (Hayano et al., 2016). These findings indicate that the trans-sulfuration pathway functions as a negative regulator in the process of ferroptosis.

Inhibition of GSH synthesis triggers ferroptosis. GSH is synthesized from glutamic acid, cysteine, and glycine, which is catalyzed by glutamate–cysteine ligase (GCL). GCL consists of a catalytic subunit, GCLC, and a modifier subunit, GCLM. The synthesis of GSH is a two-step ATP-dependent enzymatic reaction. GCL catalyzes glutamate and cysteine to convert into γ-glutamylcysteine (γ-GCS). Thereafter, γ-GCS and a molecule of glycine are catalytically converted to GSH by glutathione synthetase (GSS). GSH is a crucial antioxidant in cells and scavenges ROS (Gutierrez-Escobedo et al., 2013). The depletion of GSH results in lipid ROS accumulation, protein or membrane injury, and finally, ferroptotic cell death (Martin and Teismann, 2009). Multidrug resistance protein 1 (MRP1) prevents GSH efflux from the cells and strongly inhibits ferroptosis (Cao et al., 2019). Cisplatin, a common anti-tumor agent, could significantly reduce the level of GSH and thereby induce ferroptosis (Guo et al., 2018). In addition, abnormal synthesis of GSH could induce ferroptosis, as illustrated by the finding that inhibition of γ-GCS mediated by buthionine–sulfoximine was sufficient to trigger ferroptosis (Yang et al., 2014).

Inhibition of GPX4 induces ferroptosis. GPX4 is a peroxidase that converts GSH into oxidized glutathione (GSSH) and decreases the cytotoxic L-OOH to the corresponding harmless alcohol in membrane lipid bilayers. Hence, the inactivation or loss of GPX4 due to genetic inhibition or inhibitors or GSH depletion could result in L-OOH accumulation, triggering ferroptotic cell death (Yang et al., 2014). A study reported that the mice with the GPX4 gene knocked-out died of acute renal failure (Friedmann Angeli et al., 2014). GPX4, a seleno-protein, has seleno-cysteine as its active site (Bridges et al., 2012), which is the direct target of the canonical ferroptotic inducer RSL3 (Yang et al., 2014; Yang et al., 2016). In addition, the synthetic small-molecule inducers ML162 (or DPI7), ML210 (or DPI10), the approved anticancer agent altretamine, and the new inducer FIN56 could directly act on GPX4 and suppress GPX4 activity (Weiwer et al., 2012; Yang et al., 2014; Woo et al., 2015; Shimada et al., 2016). The genetic code of the active site seleno-cysteine is UGA, which is the same as the termination codon, thus requiring special seleno-cysteine-tRNA (Sec-tRNA) transport (Friedmann Angeli and Conrad, 2018). The maturation of Sec-tRNA is regulated by the intermediate isopentenylpyrophosphate (IPP) and CoQ10 from the mevalonate (MVA) pathway (Moosmann and Behl, 2004). Therefore, MVA pathway inhibitors (statins) could block the maturation of Sec-tRNA, subsequently affecting GPX4 synthesis and inducing ferroptosis (Chen et al., 2021a).

### 2.2 The iron metabolism axis

Iron is an essential trace element in the human body, mainly in the form of divalent iron (Fe^2+^) and trivalent iron (Fe^3+^). As a cofactor of some enzymes, iron is involved in multiple metabolic processes, such as the synthesis of ferroheme, oxygen transport, electron transport, cell proliferation, and DNA synthesis. The body maintains the stability of iron content by means of food absorption and iron circulation. Excessive absorption or an imbalance of iron circulation can easily lead to iron overload, which will contribute to oxidative damage and even cell death due to iron’s ability to accept and donate electrons (CC, 1995).

Most mammalian cells uptake iron (Fe^2+^) mainly in two ways: intestinal absorption and senescent erythrocyte degradation. In the serum, Fe^2+^ is oxidized by ceruloplasmin to Fe^3+^ and then binds with transferrin (TF) to form a TF–Fe^3+^ complex, which is endocytosed through the cell membrane transferrin receptor 1 (TFR1) (Frazer and Anderson, 2014). In the endosome, Fe^3+^ is reduced to Fe^2+^, and the latter is transported out by the divalent metal ion transporter 1 (DMT1) or the zinc-iron-regulatory protein family 8/14 (ZIP8/14) (Bogdan et al., 2016). Subsequently, Fe^2+^ is involved in the unstable iron pool (LIP). Ferritin is the major intracellular iron storage protein, which is composed of ferritin light chain (FTL) and ferritin heavy chain 1 (FTH1). Ferritin could be degraded in lysosomes by ferritinophagy to increase free iron levels (Gammella et al., 2015; Hou et al., 2016). In addition, FTH1 functions as ferroxidase, oxidizing Fe^2+^ to Fe^3+^. Because the oxidation–reduction of Fe^2+^ to Fe^3+^ requires electron transfer, iron is involved in the electron migration between substrates (Bennett and Gralnick, 2019). In 1894, Fenton first reported the Fenton reaction between Fe^2+^ or Fe^3+^ ions and hydrogen peroxide (H_2_O_2_) to produce highly reactive hydroxyl radicals (Fenton, 1894), which increased ROS level in cells and triggered the peroxidation of polyunsaturated fatty acids (PUFAs) or saturated fatty acids in membrane lipids (Lachaier et al., 2014). In addition, Fe^2+^ is a cofactor of lipoxygenases (LOXs) that catalyzes PUFA-containing phospholipids into pro-ferroptotic lipid peroxidation (Yang et al., 2016). Furthermore, intracellular Fe^2+^ is pumped into the extracellular space through membrane ferritin 1 (SLC40A1/FPN1) to strictly maintain cellular iron homeostasis (Bogdan et al., 2016). In general, the dysregulation of iron metabolism-related proteins and an increase of the active iron pool to an iron overload state can induce ferroptosis.

Many studies have demonstrated that iron metabolism-related proteins could be used as targets to regulate ferroptosis. The combination of the lysosome disruptor siramesine and the tyrosine kinase inhibitor lapatinib increased the expression of TF and reduced the expression of FPN1 in breast cancer cells, thereby promoting iron overload in the lysosome and ultimately inducing ferroptosis (Ma et al., 2016). Heat shock protein β-1 (HSPB1) suppressed TFR1 expression and downregulated TFR1-mediated iron uptake by stabilization of actin cytoskeleton, further reducing intracellular iron concentrations. Thus, the overexpression of HSPB1 could inhibit ferroptosis (Chen et al., 2006; Sun et al., 2015). Furthermore, iron-regulatory proteins (IRPs), comprising IRP1and IRP2, regulated the expression of TF, TFR1, DMT1, and FPN1 by binding to a stem-loop structure located in the 3′- or 5′-untranslated region of the target mRNA for stabilization (Wang and Pantopoulos, 2011). Nuclear receptor coactivators 4 (NCOA4) mediated the autophagic degradation of ferritin in lysosomes, resulting in the release of free iron from ferritin to promote ferroptosis (Mancias et al., 2014). In addition, the main transcription factor of iron metabolism, iron response element binding protein 2 (IREB2), could enhance the FTL and FTH1 expression and decrease ferrous ion concentrations, thereby inhibiting erastin-induced ferroptosis (Gammella et al., 2015).

Ferroptosis could be induced by directly supplementing the cell's active iron pool to establish an iron overload state. Supplementation of ferric ammonium citrate induced the death of human fibrosarcoma HT-1080 cells. That cell death was effectively inhibited by two ferroptosis inhibitors, ferristatin-1 and liproxstatin-1 (Fang et al., 2018). Some researchers have successfully induced ferroptosis of neuroblastoma cells with ammonium ferrous sulfate (Hassannia et al., 2018). Heme oxygenase-1 (HO-1) is an important source of intracellular iron that accelerates erastin-mediated ferroptotic cell death by provision of iron (Kwon et al., 2015).

### 2.3 Lipid metabolism

The trigger of ferroptosis is lipid peroxidation accumulation in the cell membrane. When the membrane lipid was peroxided, the physical properties of lipid bilayers were altered: membrane fluidity was reduced, the ion gradient was destroyed, membrane permeability was elevated, and lateral diffusion was retarded (Catala and Diaz, 2016; Feng and Stockwell, 2018). Lipid peroxidation has toxic effects on cancer cells by two mechanisms: one is further decomposition of peroxide phospholipids into active substances, depleting nucleic acid and protein and then leading to cell iron poisoning (Kagan et al., 2017); the other is that excessive lipid peroxidation leads to the thinning and the increased curvature of the membrane, resulting in membrane instability and micelle formation, eventually causing ferroptotic cell death (Agmon et al., 2018).

PUFAs in the phospholipids (PLs) of cell membranes, rather than mono-unsaturated FA, cholesterol, and cardiolipin, are highly prone to lipid peroxidation (Yang et al., 2016). Among the PLs related to PUFAs, phosphatidylethanolamines (PEs) containing arachidonic acid (AA) or its derivative adrenic acid (AdA) have been found to be the key substrates for oxidation in ferroptosis (Kagan et al., 2017). Two key enzymes, acyl-CoA synthetase long-chain family member 4 (ACSL4) and lysophosphatidylcholine acyltransferase 3 (LPCAT3), are involved in the biosynthesis and remodeling of PEs, activation of PUFAs, and insertion of PUFAs into membrane PLs (Doll et al., 2017). ACSL4 could convert AA and AdA into AA CoA and adrenal CoA, respectively, thus participating in the synthesis of membrane phospholipids (Dixon et al., 2015; Doll et al., 2017; Kagan et al., 2017), while LPCAT3 preferentially catalyzes the insertion of acylated AA into membrane phospholipids (Dixon et al., 2015). Therefore, inhibition of the ACSL4 and LPCAT3 expression decreased the intracellular accumulation of lipid peroxide substrates, thus suppressing ferroptosis (Doll et al., 2017; Kagan et al., 2017). In renal clear cell carcinoma, HIF2α stimulated the specific enrichment of PUFAs in an ACSL4-dependent manner and promoted the sensitivity to ferroptosis (Zou et al., 2019). In addition, lipoxygenase (LOX) could oxygenate PUFA-PE to produce fatty acid hydroperoxides, thus driving cell ferroptosis (Kagan et al., 2017).

## 3 The metabolites of TCM drugs targeting ferroptosis for cancer therapy

### 3.1 Alkaloids

Solasonine, a steroidal glycoalkaloid derived from *Solanum nigrum* L. [Solanaceae*; S. nigrum fruits*]*,* presents anti-tumor effects that are related to ferroptosis (Table 1). Solasonine induced iron overload, mitochondrial injury, and the destruction of the glutathione redox system by inhibiting the expression of GPX4 and SLC7A11 in lung adenocarcinoma cells (Zeng et al., 2022). Solasonine also contributed to the ferroptosis of hepatocellular carcinoma cells by the destruction of the glutathione redox system with increasing lipid ROS levels as well as significantly decreasing the levels of GPX4, GSS mRNA, and protein (Jin et al., 2020). In addition, solasonine inhibited pancreatic cancer progression. Further research has revealed that solasonine suppressed TFAP2A protein expression by combining with Lys257, Arg255, ala256, and gly250 of TFAP2A via a hydrogen bond. TFAP2A could bind to the promoter region of the de-ubiquitination enzyme OTUB1, thus enhancing OTUB1 expression. In this case, solasonine enhanced the degradation of OTUB1-mediated SLC7A11 via TFAP2A, activating ferroptosis in pancreatic cancer (Liang et al., 2022). In short, the inhibition of the GPX4–GSH axis is responsible for the effect of solasoine on cancers.

**TABLE 1:** The metabolites from TCM targeting ferroptosis in animal models or cells.

Classifications	Metabolites	Structure	Cancer type	Model/Cell (dosage)	Related molecular mechanisms	Refs
Alkaloids	Solasonine	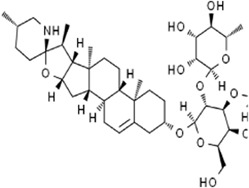	Lung adenocarcinoma Hepatocellular carcinoma Pancreatic cancer	Calu-1 and A549 cells (20 μM, 25 μM, and 30 μM) HepG2 and HepRG cells (15 ng/mL) HepG2 xenograft model (50 mg/kg/d) PANC-1 and CFPAC-1 cells (25 μM and 50 μM)	Inhibition of GPX4 and SLC7A1 expression	Jin et al. (2020), Liang et al. (2022), Zeng et al. (2022)
	Piperlongumine	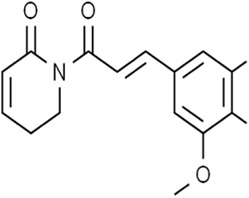	Colon cancer Hepatocellular carcinoma Breast cancer Pancreatic cancer	HT29 and SW620 cells (2.5.5 μM) HepG2, Huh7, and LM3 cells (10 μM and 20 μM) MCF-7 cells (5 μM)	Increased Nrf-2-mediated HO-1 expression	Chen et al. (2015), Lee et al. (2015), Basak et al. (2016), Yamaguchi et al. (2018)
	Trigonelline	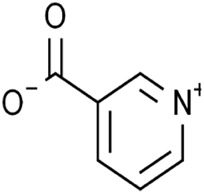	Head and neck cancer	SNU-1041, -1,066, and -1,076 cells (0.15 mM or 0.3 mM) Cisplatin-resistant HNC and SNU cells (100 μM) HN9 cell xenograft model (50 mg/kg/d)	Inhibition of the Nrf2-ARE pathway	Roh et al. (2017), Shin et al. (2018)
	Sanguinarine	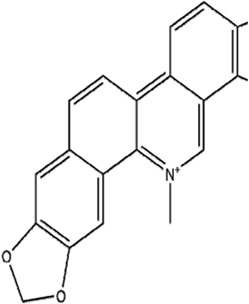	Non-small cell lung cancer	A549 and H3122 cells (10 μM) A549 cells xenograft model (5 mg/kg/d)	Decreased GPX4 expression via STUB1-mediated ubiquitination and degradation	Xu et al. (2022)
Terpenoids	Artemisinin and active metabolite Dihydroartemisinin	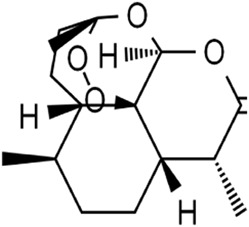	T-cell leukemia/lymphoma Renal cell carcinoma Pancreatic cancer	HTLV-1-infected T-cell lines (0–10 μM) Sunitinib-resistant KTCTL-26 cell (50 μM) Panc-1 cells (50 μM)	Decreased GSH and GPX4 expression, lysosomal iron-dependent pathway	Eling et al. (2015), Ishikawa et al. (2020), Markowitsch et al. (2020)
		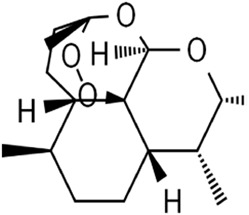	Lung cancer Hepatocellular carcinoma Head and neck carcinoma Leukemia	HEP-2 and CNE-1 cells (18 μM) NCI-H23 and XWLC-05 cells (40 μM and 60 μM) Huh-7 and HepG2 cells (20 mg/kg/d and 40 μM) Huh-1 xenograft model (7 mg/kg/d,14 mg/kg/d, and 28 mg/kg/d) HT1080 cells (20 μM) HL60, KG1, and THP-1 (10 μM) HL60 xenograft model (50 mg/kg/d)	Decreased SLC7A11, GSH, and GPX4 expression, elevated cellular free iron pool	Lin et al. (2016), Du et al. (2019), Chen et al. (2020a), Yuan et al. (2020), Su et al. (2021)
	β-elemene (treated with cetuximab)	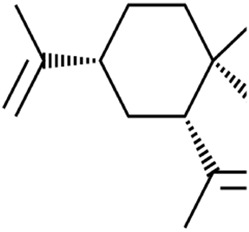	Colorectal cancer	KRAS-mutant CRC cell HCT116 and Lovo (125 μg/mL) HCT116 xenograft model (50 mg/kg/d)	Increased HO-1 and transferrin expression, decreased GPX4, SLC7A11, FTH1, and SLC40A1 expression	Chen et al. (2020b)
	Curcumenol	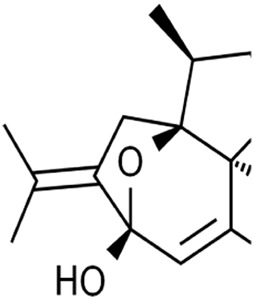	Lung cancer	H1299 and H460 cells (300 μg/mL) H460 xenograft model (200 mg/kg/d)	lncRNA H19/miR-19b-3p/FTH1 axis	Zhang et al. (2022b)
	Cryptotanshinone	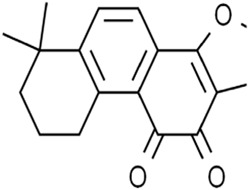	Lung cancer Liver cancer	A549 and NCI-H520 cells (40 μM) HepG2 cell (93.73 μM)	Decreased GPX4 activity/level, increased iron load	Liu et al. (2021b), Li et al. (2021)
	DihydroisotanshinoneⅠ	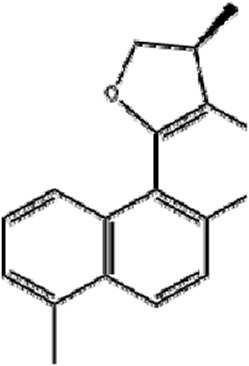	Breast cancer	MCF-7 and MDA-MB-231 cells (10 μM) MCF-7 cells xenograft model (30 mg/kg/2d) A549 and H460 cells (30 μM)	Inhibition of GPX4 expression	Lin et al. (2019), Wu et al. (2021)
	TanshinoneⅡA	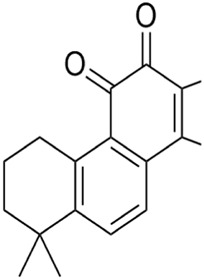	Gastric cancer	BGC-823 and NCI-H87 cells (2 μM and 4 μM) BGC-823 cell xenograft model (50 mg/kg/d)	Downregulated p53-mediated SLC7A11 expression	Guan et al. (2020)
	Celastrol (treated with erastin)	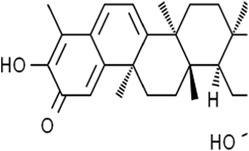	Lung cancer	HCC827, A540, and H1299 cells (1.25 μM) HCC827 cell xenograft model (1 mg/kg/d)	Elevated ROS level, disrupted mitochondrial membrane potential	Liu et al. (2021b)
	Cucurbitacin B	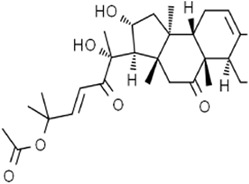	Nasopharyngeal cancer	CNE1 cells (50 μM) CNE1 cell xenograft model (0.5 mg/kg/d and 1 mg/kg/d)	Decreased GPX4 and GSH expression, accumulation of iron	Huang et al. (2021)
	Oleanolic acid	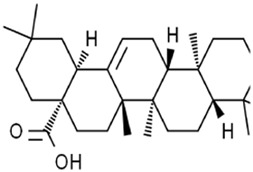	Cervical cancer	Hela cells (20 μM) Hela cell xenograft model (40 mg/kg/d and 80 mg/kg/d)	Increased ACSL4 expression	Xiaofei et al. (2021)
	Ursolic acid (treated with erastin)	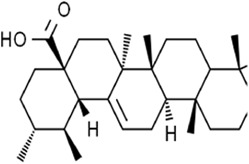	—	Various human cancer cells (6 μM) HCT116 cell xenograft model (200 mg/kg/2d)	Decreased SLC7A11 level	Li et al. (2022b)
Flavonoids	Baicalein	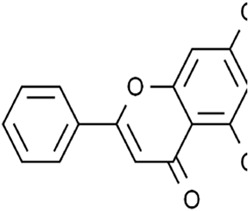	Pancreatic ductal adenocarcinoma Acute lymphoblastic leukemia	PANC1 and BxPc3 cells (10 μM) Acute lymphoblastic leukemia (ALL) cells (5 μM)	Decreased erastin-induced ferrous iron level and erastin-mediated degradation of GPX4	Perez et al. (2009), Xie et al. (2016), Probst et al. (2017)
	Robustaflavone A	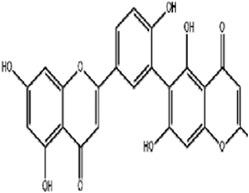	Breast cancer	MCF-7 cells (5 μM and 10 μM)	Decreased E3 ubiquitin ligase NEDD4 expression	Xie et al. (2021)
	Gambogenic acid (treated with 5 ng/mL)	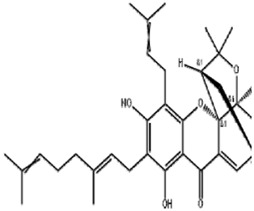	Melanoma	A375 and A2058 cells (1 μM, 2 μM, and 4 μM)	Activated p53/SLC7A11/GPX4 pathway	Wang et al. (2020)
	Ginkgetin (treated with cisplatin)	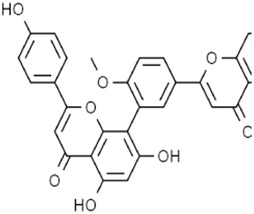	Non-small cell lung cancer	A549 cells (5 μM) A549 cell xenograft model (30 mg/kg/d)	Decreased SLC7A11 and GPX4 expression, increased labile iron pool	Lou et al. (2021)
Saponin	Ophiopogonin B	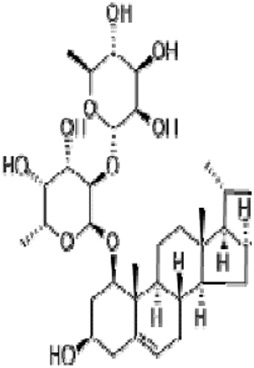	Gastric cancer Non-small cell lung cancer	AGS and NCI-N87 cells (10 μM or 20 μM) AGS cell xenograft model (50 mg/kg/d) A549 cells (5 μM) A549 cell xenograft model (2.5 mg/kg/d)	Decreased GPX4, SLC7A11, FTL, and FTH1 expression	Li et al. (2022a), Zhang et al. (2022a)
	Ginsenoside Rh4	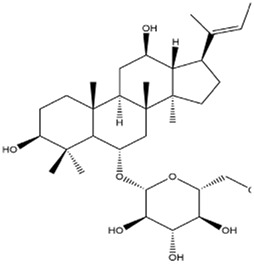	Colorectal cancer	HT29 and HCT116 cells (5 μM, 100 μM, and 200 μM) HT29 or HCT116 xenograft model (40 mg/kg/d)	Activated ROS/p53 pathway	Wu et al. (2022)
	Ardisiacrispin B	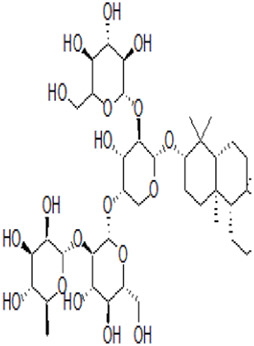	Leukemia	CCRF-CEM leukemia cells (50 μM)	Increased ROS level	Mbaveng et al. (2018b)
	Typhaneoside	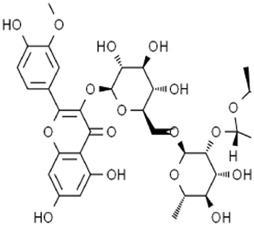	Leukemia	Kas-1, HL60, NB4, and K562 cells (20 μM, 30 μM, and 40 μM) HL60 cell xenograft model (10 mg/kg/d, 20 mg/kg/d, and 30 mg/kg/d)	Autophagy-dependent degradation of ferritin by activating the AMPK pathway	Zhu et al. (2019)
Polyphenols	Amentoflavone	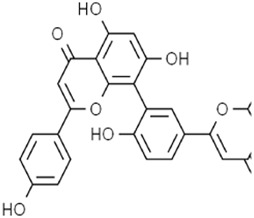	Endometrial cancer	KLE cells (50 μM, 75 μM, and 100 μM) U251 and U373 cells (10 μM and 20 μM) U251 cells xenograft model (40 mg/kg/d and 80 mg/kg/d)	Decreased SLC7A11, GPX4 and FTH1 expression and increased ACSL4 expression by activating the ROS/AMPK/mTOR pathway	Chen et al. (2020d), Sun et al. (2022)
	Gallic acid (treated with low-level laser irradiation)	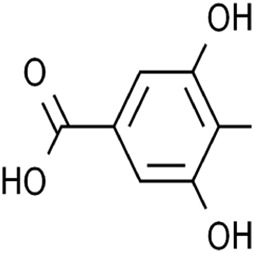	Breast cancer Melanoma	HeLa, H446, and SH-SY5Y cells (50 μg/mL) MDA-MB-231 and A375 cells (25 μg/mL)	Decreased GPX4 activity	Tang and Cheung (2019), Khorsandi et al. (2020)
	Honokiol	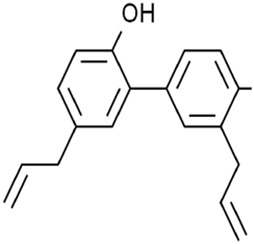	Colon cancer Acute myeloid leukemia	SW480 and RKO cells (1 μM, 10 μM, and 30 μM) RKO cell xenograft models (0.5 mg/kg/w) THP-1, U-937, and SKM-1 (15 μM and 20 μM)	Decreased GPX4 activity, upregulated HO-1 expression	Guo et al. (2021), Lai et al. (2022)
	Curcumin	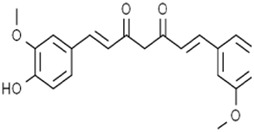	Breast cancer	MCF-7 and MDA-MB-231 cells (40 μM and 50 μM)	Upregulated HO-1 expression	Li et al. (2020a)
	6-Gingerol	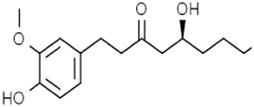	Lung cancer	A549 cells (20 μM, 40 μM, and 80 μM) A549 cell xenograft models (0.25 mg/kg/d and 0.5 mg/kg/d)	Increased autophagosomes by inhibiting USP14 expression	Tsai et al. (2020)
Polysaccharide	Red ginseng polysaccharide	—	Lung cancer Breast cancer	A549 and MDA-MB-231 cells (200 μg/mL)	Downregulated GPX4 expression	Zhai et al. (2022)
	Scutellaria barbata	—	Hepatocellular carcinoma	SMMC-7721 (44.26 mg/mL) HepG2 (42.19 mg/mL) and Huh7 cells (52.01 mg/mL) HepG2 and Huh7 cell xenograft models (14 mg/kg/d)	Decreased GPX4 and SLC7A11 expression	Li et al. (2022b)
Quinones	Juglone	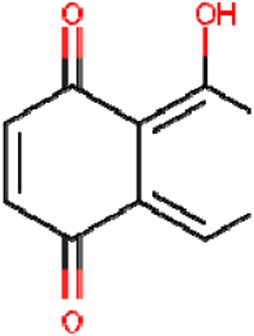	Endometrial carcinoma	Ishikawa cells (15 μM and 20 μM)	Downregulated GPX4 expression, Upregulated HO-1 expression, ferritin phagocytosis	Zhang et al. (2021)
	Physcion 8-O-β-glucopyranoside	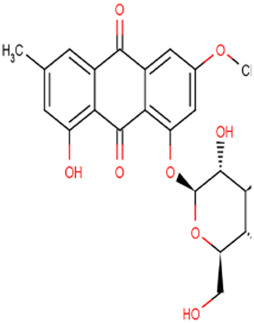	Gastric cancer	MGC-803 and MKN-45 cells (20 μM) MGC-803 cell xenograft models (30 mg/kg/d or 50 mg/kg/d)	Decreased GLS2 expression by miR-103a-3p	Niu et al. (2019)
	Plumbagin	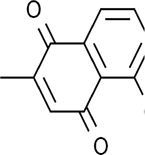	Glioma	Various glioma g/kg μM cells (4 μM)	Decreased xCT expression, increased the lysosome-dependent GPX4 degradation	Zhan et al. (2022)

Annotations: using cells as research subjects indicates *in vitro*; using xenograft models as research subjects indicates *in vivo*.

Piperlongumine (PL) is a pyridine alkaloid from *Piper longum* L. [Piperaceae*; P. longum fruit*]*.* PL significantly increased ROS levels and protein glutathionylation with a concomitant elevation in Nrf-2 expression, thus inducing the redox imbalance to kill two human colon cancer cell lines with mutant p53, HT29 and SW620 (Basak et al., 2016). Additionally, PL selectively destroyed hepatocellular carcinoma cells rather than normal hepatocytes via ROS–endoplasmic reticulum (ER)–MAPK–CHOP axis, thus significantly suppressing hepatocellular carcinoma cell migration and invasion (Chen et al., 2015). Similarity, PL selectively killed human breast cancer MCF-7 cells instead of human MCF-10A breast epithelial cells. Mechanically, PL directly interacted with Kelch-like ECH-associated protein-1 (Keap1), which resulted in Nrf-2-mediated HO-1 expression (Lee et al., 2015). HO-1 is a critical source of intracellular iron. The above results suggest that PL may induce ferroptosis. This hypothesis that PL induced human pancreatic cancer cell death mainly via the induction of ROS-mediated ferroptosis, on the grounds that the cell death could be suppressed by ferroptosis inhibitors (ferrostatin-1 and liproxstatin-1) and the iron chelator deferoxamine (DFO) (Yamaguchi et al., 2018). Furthermore, the triple combined treatment with PL, cotylenin A (CN-A; a plant growth regulator), and sulfasalazine (SSZ, a clinically approved ferroptosis inducer) was highly effective against pancreatic cancer but was not responsive to mouse embryonic fibroblasts (MEFs) (Yamaguchi et al., 2018). In general, PL is expected to reduce chemotherapeutic drug-induced side effects in clinic.

Trigonelline (TRG) is a plant alkaloid in *Trigonella foenum-graecum* L. [Fabaceae*; T. foenum-graecum dried ripe seed*]. TRG sensitized chemo-resistant head and neck cancer (HNC) cells to *in vitro* and *in vivo* RSL3 treatment by decreasing the activity of the Nrf2-ARE pathway (Shin et al., 2018). TRG also overcame the resistance of cisplatin-resistant HNC cells to artesunate-induced ferroptosis *in vitro and in vivo* by the inhibition of the Nrf2-ARE pathway (Roh et al., 2017). In a nutshell, TRG overcomes cancer resistance to chemotherapies through the inhibition of the NRF2-ARE pathway. Thus, TRG is expected to break the dilemma of tumor drug resistance and improve chemotherapy efficacy.

Sanguinarine (SAG) is a natural benzophenanthridine alkaloid from *Sanguinaria Canadensis* L. [Papaveraceae*; Sanguinaria Canadensis radix et rhizome*]. SAG contributed to ferroptosis by increasing the Fe^2+^ concentration and ROS levels and decreasing GSH levels, thus repressing the growth and metastasis of non-small cell lung cancer (NSCLC). Further research regarding the mechanism demonstrated that SAG enhanced the expression of E3 ubiquitin ligases STUB1 and led to the subsequent ubiquitination and degradation of GPX4 (Xu et al., 2022).

### 3.2 Terpenoids

Artemisinin (ART) is a natural sesquiterpene lactone extracted from *Artemisia annua* L. [Asteraceae *; A. annua dried aerial parts*], known as an anti-malarial drug. In adult T-cell leukemia/lymphoma (ATLL), ART induced intracellular ROS- and iron-dependent cytotoxicity, which was partly inhibited by treatment with an iron chelator or ferroptosis inhibitor (Ishikawa et al., 2020). ART significantly reduced GSH content and GPX4 expression in sunitinib-resistant KTCTL-26 renal cell carcinoma cells to induce ferroptosis, thus exhibiting more potent anti-tumor effects (Markowitsch et al., 2020). Likewise, ART specifically triggered ROS- and lysosomal iron-dependent cell death in pancreatic cancer cell (PDAC) lines (Eling et al., 2015). Thus, ART functions as a specific activator of ferroptosis. Dihydroartemisinin (DHA), the main active metabolite of ART, also has anti-tumor activity by inducing ferroptosis. DHA downregulated the SLC7A11 expression and reduced the GSH and GPX4 levels along with increasing the cellular lipid ROS level in lung cancer cells, hepatocellular carcinoma cells, and head and neck carcinoma cells (Lin et al., 2016; Yuan et al., 2020; Su et al., 2021). Moreover, DHA enhanced the degradation of ferritin and elevated the free iron pool to regulate iron homeostasis in a lysosome and autophagy manner by regulating the activity of the AMPK/mTOR/p70S6k signaling pathway in leukemia cells (Du et al., 2019; Chen G. Q. et al., 2020). Furthermore, DHA was associated with free iron to provoke the binding of iron-regulatory proteins (IRPs) with mRNA molecules containing an iron-responsive element (IRE) sequence, which further increased cellular free iron (Chen G. Q. et al., 2020). One study reported that DHA upregulated the PEBP1 expression by inhibiting its ubiquitination degradation, thus promoting the formation of PEBP1/15-LO (lipoxygenase) and cell membrane lipid peroxidation, finally inducing ferroptosis of hepatocellular carcinoma (Su et al., 2021). In brief, ART and DHA induce ferroptosis through a multipronged mechanism.

β-elemene is a sesquiterpene metabolite extracted from *Curcuma longa* L. [Zingiberaceae*; C. longa rhizome*]. Combinative treatment of β-elemene and cetuximab could induce ferroptosis of KRAS-mutant colorectal cancer cells (CRC) by iron-dependent ROS accumulation, GSH depletion, lipid peroxidation, upregulation of HO-1 and transferrin, and downregulation of negative regulatory proteins for ferroptosis (GPX4, SLC7A11, FTH1, glutaminase, and SLC40A1), thus inhibiting tumor growth and lymph node metastases *in vivo* (Chen et al., 2020b). Curcumenol, a sesquiterpenoid metabolite, is an important monomer extracted from *Curcuma zedoaria* [Zingiberaceae*; C. zedoaria rhizome*]. Curcumenol induced ferroptosis of lung cancer cells via the lncRNA H19/miR-19b-3p/FTH1 axis (Zhang L. et al., 2022).

Cryptotanshinone (CTN) is a quinoid diterpene extracted from *Salvia miltiorrhiza Bunge* [Lamiaceae*; Salviae miltiorrhizae radix rhizome*]. CTN decreased GPX4 activity and ferroportin levels and increased iron load, which induced iron-dependent lipid peroxidation. Thus, CTN triggered ferroptosis in lung cancer cells (Li et al., 2021). In addition, CTN induced ferroptosis of human liver cancer HepG2 cells by significantly enhancing ROS accumulation, reducing GSH level, and downregulating the xCT and GPX4 expression, which could be inhibited by ferrostatin-1 (Fer-1, ferroptosis inhibitor) (Liu JL. et al., 2021). DihydroisotanshinoneⅠ(DT) is a diterpenoid metabolite in *S. miltiorrhiza*. DT induced ferroptosis and apoptosis of breast and lung cancer cells by repressing GPX4 expression, thus significantly inhibiting tumor growth without adverse effects in a xenograft nude mouse model (Lin et al., 2019; Wu et al., 2021). TanshinoneⅡA (Tan ⅡA) is another natural lipophilic diterpene isolated from *S. miltiorrhiza*. Tan ⅡA induced ferroptosis by P53-mediated SLC7A11 downregulation to inhibit the proliferation of gastric cancer (Guan et al., 2020). In brief, terpenoids extracted from *S. miltiorrhiza* regulate the system Xc^−^-GSH-GPX4 axis to induce ferroptosis.

Celastrol is a triterpene extracted from *Tripterygium wilfordii Hook. F.* [Celastraceae*; T. wilfordii root].* The combination of celastrol with erastin significantly elevated ROS levels, disrupted mitochondrial membrane potential, and promoted mitochondrial fission. Thus, co-treatment with celastrol and erastin induced NSCLC ferroptosis by activating the ROS–mitochondrial fission–mitophagy axis in an HSF1-dependent manner (Liu JL. et al., 2021). Cucurbitacin B (CuB) is a triterpenoid molecule isolated from *Trichosanthes kirilowii Maxim.* [Cucurbitaceae*; T. kirilowii dried root]*. CuB incited the accumulation of iron and GSH depletion, subsequently downregulating GPX4 expression to increase lipid peroxidation and eventually triggering cell death of human nasopharyngeal cancer cells in the form of ferroptosis. Therefore, CuB markedly inhibited tumor progression without obvious side reactions *in vivo* (Huang et al., 2021).

Oleanolic acid (OA) is a pentacyclic triterpene widely distributed in the leaves, fruits, and seeds of Oleaceae plants, such as *Olea europaea* L. [Oleaceae*; O. europaea leave and fruit*] and *Ligustrum lucidum W.T.Aition* [Oleaceae*; L. lucidum fruit]*. OA resulted in the accumulation of Fe^2+^ and elevated the ferroptosis-related protein and ACSL4 expressions to activate the ferroptosis of cervical cancer HeLa cells, thus suppressing cancer cell proliferation. In knocked-down ACSL4, the anticancer effect of OA was canceled, and the levels of ROS and GPX4 were decreased, suggesting that OA triggered ferroptosis by promoting the expression of ACSL4 (Xiaofei et al., 2021).

Ursolic acid (UA) is a natural pentacyclic triterpene derived from a great variety of traditional medicinal plants and most fruits and vegetables. Sorafenib/UA induced ferroptosis in various human cancer cells by decreasing the level of SLC7A11 and inciting dramatic accumulation of intracellular ROS. In addition, this combination treatment also induced apoptosis by promoting myeloid cell leukemia-1 (Mcl-1) degradation (Li L. et al., 2022). Therefore, UA boosted the anti-tumor activity of sorafenib in tumor xenograft models.

### 3.3 Flavonoids

Baicalein is a flavonoid active metabolite extracted from the root of *Scutellaria baicalensis Georgi* [Lamiaceae*; S. baicalensis root]*. A previous study showed that baicalein could decrease the yield of thiobarbituric acid, oxygen consumption rate, and iron reduction rate in the reaction system of ascorbic acid and FeCl_3_, and it showed a similar effect with iron chelators. Baicalein was combined with the microsomal membrane to inhibit lipid peroxidation through the formation of the iron–baicalein complex (Gao et al., 1996). Subsequent research revealed that baicalein decreased erastin-induced ferrous iron levels and erastin-mediated degradation of GPX4, GSH depletion, and lipid peroxidation in the human pancreatic ductal adenocarcinoma cell lines PANC1 and BxPc3 (Xie et al., 2016). The O6/O7 oxygen atoms of the A-ring on baicalein serve as the iron-binding site. Baicalein also strongly inhibited the Fe-promoted Fenton chemistry via a combination of chelation and radical scavenging mechanisms under physiologically relevant conditions (Perez et al., 2009). In acute lymphoblastic leukemia (ALL) cells, baicalein reduced lipid peroxidation and ROS production to impede RSL3-induced ferroptosis (Probst et al., 2017). Thus, baicalein is a potent inhibitor of ferroptosis and is expected to be a potential drug to prevent ferroptosis-associated tissue injury. However, a controversial result revealed that baicalein-mediated ferroptosis of bladder cancer cells was triggered by downregulating FTH1, accompanied by the accumulation of ROS and iron (Kong et al., 2021). This opposite result may be related to the cell line.

Robustaflavone A (RF-A) is a bioflavonoid isolated from *Selaginella trichoclada Alston* [Selaginellaceae *; S. trichoclada whole plant*]. RF-A enhanced VDAC2 channel level and decreased E3 ubiquitin ligase NEDD4 expression, resulting in lipid peroxidation and ROS production to trigger ferroptosis in breast cancer cells (Xie et al., 2021). It is worth emphasizing that RF-1 was isolated and purified by researchers, and the purity of this metabolite was not disclosed in this study. Gambogenic acid (GNF) is a polyprenylated xanthone extracted from the plant *Garcinia morella Desr*. GNF triggered ferroptosis in TGF-β1-treated melanoma cells by activating the p53/SLC7A11/GPX4 axis (Wang et al., 2020). Ginkgetin is a bioflavonoid derived from *Ginkgo biloba* L. [Ginkgoaceae*; G. biloba leaves and seed*]. The combination of ginkgetin and cisplatin (DDP) decreased the SLC7A11 and GPX4 expression as well as the GSH/GSSG ratio and increased labile iron pool and lipid peroxidation to mediate ferroptosis in EGFR wild-type non-small-cell lung cancer. In addition, ginkgetin significantly decreased DDP-induced elevation of the binding of Nrf2 to the HO-1 promoter, thus decreasing HO-1 nuclear expression and reversing DDP-induced HO-1 nuclear translocation, ultimately enhancing the therapeutic effect of DDP (Lou et al., 2021).

### 3.4 Saponin

Ophiopogonin B (OP-B) is a saponin extracted from *Ophiopogon japonicas (Thunb.) Ker Gawl.* [Asparagaceae *; Ophiopogon japonicus root tuber*]. OP-B decreased the expression of GPX4 and SLC7A11 but had no effect on NCOA4 and FTH1 expression, thereby inducing ferroptosis in gastric cancer *in vitro and in vivo,* thus reducing the volume and weight of tumors in tumor-bearing nude mice (Zhang R. et al., 2022). In addition, OP-B induced ferroptosis by downregulating the ferroptosis-negative regulatory Aurora kinase A (AURKA) in NSCLC (Li Y. et al., 2022).

Ginsenoside Rh4 is an active metabolite of triterpenoid saponins in *Panax ginseng C.A.Mey*. [Araliaceae*; P. ginseng root and rhizome*]. Rh4 induced ferroptosis via autophagy activation in colorectal cancer cells, which was related to the activation of the ROS/p53 signaling pathway (Wu et al., 2022). Ardisiacrispin B is a triterpenoid saponin extracted from *Ardisia staudtii Gilg [*Primulaceae*; A. staudtii fruit]*. Ardisiacrispin B showed cytotoxic effects on multi-factorial drug-resistant cancer cells via increased ROS-mediated ferroptosis and apoptosis (Mbaveng et al., 2018b). Typhaneoside (TYP) is a main flavonoid in the extract of *Typha angustifolia* L. [Typhaceae*; T. angustifolia dried pollen*]. TYP incited the autophagy-dependent degradation of ferritin and ROS accumulation by activating the MP-activated protein kinase (AMPK) signaling pathway and then inducing the ferroptosis of acute myeloid leukemia (AML) cells (Zhu et al., 2019).

### 3.5 Polyphenols

Amentoflavone (AF) is a polyphenolic metabolite extracted from *Selaginella tamariscina (P.Beauv.)* [Selaginellaceae *; S. tamariscina whole plant*]. AF evoked ferroptosis along with decreasing SLC7A11, GPX4, and FTH1 expression and increasing ACSL4 expression by regulating the activation of ROS/AMPK/mTOR signaling, which suppressed the viability, proliferation, and malignant progression of endometrial cancer cells (Sun et al., 2022). In addition, AF triggered autophagy-dependent ferroptosis by blocking intracellular iron trafficking and storage to destroy iron homeostasis in human glioma, which was related to the activation of AMPK/mTOR/p70S6K signaling (Chen G. Q. et al., 2020). In brief, AF induces ferroptosis by regulating the AMPK/mTOR signaling pathway.

Gallic acid (GA), a natural polyhydroxy phenolic metabolite, is identified in various edible mushrooms, fruits, and vegetables. GA caused multiple forms of cell death, such as apoptosis characterized by mitochondrial cytochrome *c* release and caspase-3 activation, ferroptosis characterized by lipid peroxidation, and necroptosis characterized by loss of plasma membrane integrity, in an iron-dependent manner (Tang and Cheung, 2019). Moreover, GA combined with low-level laser irradiation increased ROS production and decreased GPX4 activity to trigger ferroptosis and apoptosis of breast and melanoma cancer cells (Khorsandi et al., 2020).

Honokiol (HNK) is a biphenolic metabolite in *Magnolia officinalis Rehder and E.H.Wilson* [Magnoliaceae*; M. officinalis dried stem bark, root bark, and branch bark]*. In colon cancer cells, HNK increased ROS and Fe^2+^ levels by decreasing the GPX4 activity without influencing system Xc^−^ to induce ferroptosis, thus reducing cancer cell viability (Guo et al., 2021). Additionally, HNK induced the ferroptosis of acute myeloid leukemia (AML) in a non-canonical manner by significantly upregulating HO-1 expression instead of downregulating SLC7A11 expression (Lai et al., 2022). Together, the system Xc^−^ is not a target for HNK.

Curcumin is a phytopolylphenol metabolite isolated from *C. longa* L. [Zingiberaceae*; C. longa radix rhizome*]. Curcumin induced the ferroptotic death of breast cancer cells by upregulating HO-1 expression characterized by a marked accumulation of intracellular iron, ROS, lipid peroxides, and the downregulated GSH level (Li R. et al., 2020). 6-Gingerol is a phenol in *Zingiber officinale Roscoe* [Zingiberaceae*; Z. officinale dried rhizome]*. 6-Gingerol enhanced autophagy-dependent ferroptosis of lung cancer cells by decreasing the expression of autophagy-related proteins ubiquitin-specific peptidase (USP14) and increasing iron concentrations and ROS levels, thereby presenting an anti-tumor effect *in vivo and in vitro* (Tsai et al., 2020).

### 3.6 Polysaccharide

Red ginseng polysaccharide (RGP) is an active metabolite from *P. ginseng C.A.Mey [*Araliaceae*; P. ginseng radix rhizome]*. RGP promotes ferroptosis of lung and breast cancer cells by downregulating GPX4 expression (Zhai et al., 2022). Scutellaria barbata is a polysaccharide in the dried botanical drug of *Scutellaria barbata D. Don* [Lamiaceae *; S. barbata whole plant*]. *Scutellaria barbata* reduced GPX4 and SLC7A11 mRNA and protein levels to induce ferroptosis characterized by iron-mediated lipid peroxidation and ROS metabolism in hepatocellular carcinoma, thus suppressing HCC tumorigenicity *in vivo* (Li H. et al., 2022).

### 3.7 Quinones

Juglone is a naphthoquinone isolated from the green peel of *Carya cathayensis Sarg. [*Juglandaceae*; C. cathayensis green peel]*. Juglone resulted in the downregulation of GPX4 expression, GSH depletion, and the upregulation of HO-1 expression. In addition, juglone induced ferritin phagocytosis to increase Fe^2+^, which inhibited the migration and invasion of endometrial carcinoma Ishikawa cells (Zhang et al., 2021). Physcion 8-O-β-glucopyranoside (PG), an anthraquinone extracted from *Rumex japonicus Houtt.* [Polygonaceae*; R. japonicus root and fruit]*, upregulated ROS levels and intracellular Fe^2+^ levels by downregulating the inhibitory effect of miR-103a-3p on glutaminase 2 (GLS2) expression to induce ferroptosis of gastric cancer (Niu et al., 2019). GLS2 is a positive ferroptosis regulator that promotes the transformation of glutamine to glutamate in cancer cells (Li et al., 2015).

Plumbagin is a naphthoquinone substance obtained from *Plumbago zeylanica* L. [Plumbaginaceae*; P. zeylanica root*]. Plumbagin increased NAD(P)H quinone dehydrogenase 1 (NQO1) activity and decreased xCT expression, resulting in NQO1-dependent glioma cell death. It also induced the lysosome-dependent degradation of GPX4 and caused GPX4-dependent glioma cell death (Zhan et al., 2022).

### 3.8 Miscellaneous

Erianin is a dibenzyl metabolite isolated from *Dendrobium chrysotoxum Lindl.* [Orchidaceae*; D. chrysotoxum stem]*. Erianin induced ferroptotic lung cancer cell death via Ca^2+^/CaM signaling accompanied by ROS accumulation, lipid peroxidation, and GSH depletion and increased HO-1 and transferrin levels, thus suppressing cancer cell growth and migration (Chen et al., 2020c). In bladder cancer cells, erianin evoked ferroptosis by inducing NRF2 inactivation characterized by decreased FTH1, GPX4, HO-1, and xCT/SLC7A11 expression as well as the accumulation of ROS and the depletion of GSH (Xiang et al., 2021).

Epunctanone, a benzophenone metabolite from *Garcinia epunctata Stapf* [Clusiaceae *; G. epunctata whole plant*], induced ferroptosis and apoptosis of multidrug-resistant cancer cells by increasing the production of ROS (Mbaveng et al., 2018a). The metabolite of the natural product parthenolide, DMOCPTL, induced ferroptosis via ubiquitination of GPX4 in triple-negative breast cancer cells (TNBC), thus effectively inhibiting breast tumor growth (Ding et al., 2021).

Alloimperatorin (AM) is a metabolite derived from *Angelica dahurica (Hoffm.)* Benth. & Hook.f. ex Franch. & Sav.[Apiaceae *; Angelica dahurica root*]. AM resulted in remarkable mitochondrial shrinkage, enhanced Fe^2+^ and ROS levels, and significantly decreased mRNA and protein expression of SLC7A11 and GPX4 in breast cancer cells, indicating that AM suppressed cell growth and invasion via ferroptosis (Zhang J. et al., 2022). Bufotalin (BT) is a steroid lactone isolated from *Venenum bufonis*. BT caused lipid peroxidation in NSCLC by accelerating the ubiquitination-dependent degradation of GPX4 and increasing the intracellular Fe^2+^ level (Zhang W. et al., 2022). Agrimonolide is isolated from *Agrimonia pilosa Ledeb.* [Rosaceae*; A. pilosa dried aerial parts].* It directly inhibited the stearoyl-CoA desaturase-1 (SCD1) protein to trigger ferroptosis of ovarian cancer, evidenced by the increased ROS, total iron, and Fe^2+^ levels and downregulation of ferroptosis inhibitors (SLC7A11 and GPX4), thus suppressing cancer progression in the SKOV-3 xenograft model (Liu et al., 2022).

Some TCM botanical drugs could induce ferroptosis of cancer cells, but the specific metabolites have not been identified or isolated. *Betula pendula subsp. Pendula [*Betulaceae*; B. pendula subsp. Pendula radix]* bark methanolic extract enhanced an oxidative cellular microenvironment, leading to ferroptosis-mediated CaCo2 cell death by HO-1 hyper-expression (Malfa et al., 2019). *Scleromitrion diffusum (Willd.) R.J.W*a*ng [*Rubiaceae*; S. diffusum whole plant]* regulated VDAC2/3 activity by promoting BAX via repressing Bcl2 expression, thus triggering ferroptosis in lung adenocarcinoma cells (Huang J. et al., 2022). *Actinidia chinensis Planch.* (ACP) [Actinidiaceae*; A. chinensis radix*] promoted the accumulation of ROS via suppressing GPx4 and xCT (SLC7A11) proteins to trigger ferroptosis, thus preventing the proliferation and migration of gastric cancer (Gao et al., 2020).

In addition, some natural drugs promote ferroptosis of cancer cells, but the understanding of the mechanism is in its infancy. MAP30, a bioactive protein isolated from seeds of *Momordica charantia* L. [Cucurbitaceae*; M. charantia whole plant*], played a synergistic role with cisplatin in inhibiting ovarian cancer by altering metabolism and inducing ferroptosis *in vivo* (Chan et al., 2020). The chloroform extract of *Fumaria officinalis* L. [Papaveraceae *; F. officinalis whole flowering plant*] induced iron-dependent cell death in multiple myeloma cells (Adham et al., 2021). *Thymus vulgaris* L. [Lamiaceae*; T. vulgaris whole plant*] *and Arctium lappa* L. [Asteraceae*; A. lappa dried ripe fruit*] extracts inhibited cell proliferation of leukemia and multiple myeloma by inducing ferroptosis (Aveen et al., 2020). Ferroptosis partly contributed to andrographis-mediated chemosensitization in colorectal cancer (Sharma et al., 2020).

## 4 Conclusions and perspectives

Various natural pharmaceutical metabolites induce ferroptosis in cancer cells via multiple pathways and multiple targets. These studies validate that natural pharmaceutical metabolites from TCM can cause changes in ferroptosis-related proteins, but the in-depth mechanism of protein changes has not been clarified. Furthermore, the current results are obtained from cell and animal models. Considering that the pathogenesis of human and animal diseases may be completely different and animal models cannot predict the safety and efficiency of drugs on humans, clinical trials are essential for applying TCM drugs to induce ferroptosis for cancer therapy. Clinical trial studies will better discover or verify the efficacy, adverse reactions, permeability, persistence, and excretion of TCM drugs.

In addition, the significant feature of TCM drugs is that the compatibility of different Chinese botanical drugs will achieve unexpected therapeutic effects. Therefore, future research should study of the compatibility of different active metabolites to form a traditional Chinese medicine formula to treat cancer. For example, Fuzheng Jiedu Xiaoji formulation (FZJDXJ) is a mixture of the botanical drugs *Codonopsis pilosula (Franch.) Nannf. [*Campanulaceae*; C. pilosula root]*, *Astragalus mongholicus Bunge [*Fabaceae*; A. mongholicus root],* and *Angelica sinensis (Oliv.) Diels [*Apiaceae*; A. sinensis root].* It is most commonly prescribed for HCC treatment in Beijing Ditan Hospital (Yang et al., 2021). Animal experiments have shown the natural metabolites contained in FZJDXJ could regulate ferroptosis. Thus, FZJDXJ combined with ferroptosis inducers may achieve unexpected effects in clinical scenarios.

In addition, the combination therapy of TCM and current chemotherapeutic drugs may hold promise as an efficient therapy option for the selection of patients with therapy-resistant cancers or unwanted off-target effects. However, some studies have shown that excessive ferroptosis can lead to the release of damage-associated molecular patterns and the activation of immune responses triggered by ferroptotic damage, which promote tumor growth and metastasis. Precisely regulating the activation intensity of ferroptosis to inhibit cancers is an important scientific issue that needs to be addressed in future research. Additionally, it is necessary to evaluate what cancer types or patient groups are more suitable for targeting ferroptosis. Iron levels and ferroptosis-related gene expression or mutations should be combined to evaluate which patients are most likely to benefit from treatment targeting ferroptosis.
